# Aphasia Therapy in the Age of Globalization: Cross-Linguistic Therapy Effects in Bilingual Aphasia

**DOI:** 10.1155/2014/603085

**Published:** 2014-03-11

**Authors:** Ana Inés Ansaldo, Ladan Ghazi Saidi

**Affiliations:** ^1^Centre de Recherché de l'Institut Universitaire de Gériatrie de Montréal, 4565 Queen Mary Road, Montréal, QC, Canada H3W 1W5; ^2^Speech-Language Pathology and Audiology Department, Faculty of Medicine, University of Montreal, Pavillon 7077 Avenue du Parc, local 3001-1, Montréal, QC, Canada H3N 1X7

## Abstract

*Introduction*. Globalization imposes challenges to the field of behavioural neurology, among which is an increase in the prevalence of bilingual aphasia. Thus, aphasiologists have increasingly focused on bilingual aphasia therapy and, more recently, on the identification of the most efficient procedures for triggering language recovery in bilinguals with aphasia. Therapy in both languages is often not available, and, thus, researchers have focused on the transfer of therapy effects from the treated language to the untreated one. *Aim*. This paper discusses the literature on bilingual aphasia therapy, with a focus on cross-linguistic therapy effects from the language in which therapy is provided to the untreated language. *Methods*. Fifteen articles including two systematic reviews, providing details on pre- and posttherapy in the adult bilingual population with poststroke aphasia and anomia are discussed with regard to variables that can influence the presence or absence of cross-linguistic transfer of therapy effects. *Results and Discussion*. The potential for CLT of therapy effects from the treated to the untreated language depends on the word type, the degree of structural overlap between languages, the type of therapy approach, the pre- and postmorbid language proficiency profiles, and the status of the cognitive control circuit.

## 1. Introduction

### 1.1. Bilingualism Is a Distinctive Feature of Globalization

Contemporary society is characterized by a bilingual or multilingual mode of communication. Whether for historic, economic, or migration reasons, bilingualism is no longer exceptional, but most often the rule. Whereas some countries have a history of bilingual and polyglot modes of communication, the era of globalization has contributed to the promotion of bilingualism around the world. Nowadays, bilingualism provides better career opportunities in all sectors of the economy and human activity, a fact that has motivated a wider interest in second language learning. Parents are increasingly choosing bilingual education as a result of evidence suggesting that bilingual children may develop specific cognitive advantages [1, 2], including enhanced intellectual development, greater creativity and flexibility, and openness to cultural diversity. For all of these reasons, social, educational, healthcare, and political policies are expected to adapt to such multilingual and multicultural societies.

### 1.2. Bilingual Aphasia

Aphasia is an acquired language disorder resulting from brain damage. It refers to a breakdown in the ability to formulate, retrieve, or decode the arbitrary symbols of language. It is usually acquired in adulthood [3].

The bilingual population is large and growing worldwide; therefore, bilingual aphasia is becoming more and more frequent. The complexity of the behavioural patterns observed in bilingual aphasia is big, since it concerns two (or more) languages, whose recovery does not always follow equivalent patterns. Moreover, given the almost endless possible combinations of language pairs, the issue of bilingual aphasia therapy is a big challenge. Thus, even the most avant-garde educational policies aimed at training bilingual speech-language pathologists are likely to provide only partial solutions to the clinical management of this population [4, 5]. Consequently, the study of cross-linguistic-language-therapy effects is likely to become an unavoidable topic in the field of aphasiology in the years to come.

From a neurorehabilitative perspective, bilingualism imposes a certain number of challenges regarding the assessment and intervention provided to bilingual clinical populations, particularly, those that suffer from cognitive impairment. The complexity of this issue extends well beyond the linguistic knowledge required to interact with the patient so as to detect impaired language abilities. Beyond language, there is communication, that is, the ability to decode the pragmatics that characterize a specific linguistic community. This is essential for the proper understanding of communicative behavior, meaning, what is normal, and what is not, in the context of a given culture.

The issue of language impairment in bilinguals has interested cognitive neuroscientists for more than a century. In particular, the study of bilingual aphasia first focused on the variety of aphasia patterns characterizing bilingual clinical populations [5–8]. Furthermore, the development of testing procedures that take into consideration the linguistic particularities gave raise to bilingual aphasia tests for a variety of language pairs, among which the BAT [9, 10] developed for more than 59 languages and the Multilingual Aphaisa Examination developed in six languages [11], along with tests normalized in several languages, such as the Aachen Aphasia [12–14], and the Boston Diagnostic Aphasia Examination [15–18]. These tests provide a linguistically valid assessment of bilingual aphasia.

More recently, aphasiologists have focused on the complex issue of bilingual aphasia language therapy, with the purpose of developing the most efficient procedures for triggering language recovery in this population. This is a relatively new field, and a complex one, given that it requires juggling the complexities of bilingual language processing, which amounts to more than simply the additive processing of two languages.

## 2. Aims

The purpose of this paper is to discuss the literature on bilingual aphasia therapy, with a focus on the cross-linguistic effects that language therapy provided in one of the two languages of the patient may (or may not) have on the untreated language.

This paper will discuss a number of factors with CLT potential: (a) word category (cognates versus non-cognates), (b) language distance (same versus distant language families), (c) pre and post morbid proficiency in either language, and (d) the impact of cognitive control issues on transfer of therapy effects. Finally, the main clinical implications of research findings on cross-linguistic transfer of therapy effects (CLTE) in bilingual aphasia therapy will be discussed, with the purpose of proving intervention efficacy in bilingual populations with language impairment, while optimizing health care efficiency in terms of resource training and allocation. This research will contribute to intervention efficacy in bilingual populations with language impairment, while optimizing health care efficiency in terms of resource training and allocation.

## 3. Methods

The evidence discussed in this paper was collected from the following databases: Medline, ASHA, Cochrane, Aphasiology Archive, Evidence-Based Medicine Guidelines, NHS Evidence, and PsycBite et Speechbite. The key words bilingual, aphasia, cross-language, generalization, cognates, naming treatment, and transfer guided the search. This resulted in fifteen articles, two of which received the largest weight in the analysis, since they were systematic reviews [19, 20] with an A-level recommendation that witnesses for good quality patient-oriented research, according to the AFF taxonomy [21]. The remaining articles report case series, or single-case design studies whose level of evidence is much lower; however, all of these were selected because they respected a number of criteria that allowed some degree of generalization of the reported findings. Specifically, the inclusion criteria consisted the following:

- provide details on pre- and posttherapy bilingual aphasia profiles,
- describe therapy procedures in sufficient detail to make them replicable,
- provide information on therapy frequency,
- discuss a number of variables that may have influenced the presence or absence of cross-linguistic transfer effects,
- reported evidence which concerns the adult population with acquired language impairment,
- reported that patient speaks at least two Indo-European languages with different degrees of proficiency across languages before brain damage,
- focused mostly on therapy for word-retrieval deficits, namely, anomia, which constitutes the most widespread aphasia symptom across all aphasia types.

## 4. Results

### 4.1. Cross-Linguistic Effects in Bilingual, Healthy, and Brain Damaged Populations

Understanding the mechanisms that rule cross-linguistic transfer in bilingual healthy populations highlights the functioning of the bilingual language system.

There is convergent evidence on the fact that the speech of a bilingual person reflects the influence of one language on the other [22, page 5]. This influence, which results from similarities and differences between the target language and any other previously acquired language, is referred to as* cross-linguistic influence* or* cross-linguistic transfer* (CLT) [22, page 27]. Similarities and differences can be observed at different levels of language processing, namely, the word level, the syntax, and phonology levels, as well as the proficiency level. Thus, the study of CLT effects among healthy bilinguals provides clues about the mechanisms that rule CLTE, some of which have been exploited in bilingual aphasia therapy.

#### 4.1.1. Word Type: Cognates, Clangs, and Noncognates

There is extensive evidence on CLT effects with cognates and clangs, as opposed to noncognates [23, 24]. Cognates are formally equivalent words whose meanings may be identical or almost so [25, page 73] (e.g., “tiger” (/′tīg*ə*r/) and “*tigre*” (/tigr/)), whereas clangs (or homophones) are phonologically similar words with different meanings (e.g., “bell” /b*ɛ*l/; metal object that makes a ringing sound when struck; Sonnette in French) in English and “*belle*” in French (/b*ɛ*l/; meaning beautiful). Finally, noncognates are translation equivalents that share semantics but not phonology, such as “butterfly” in English and its Spanish equivalent, “*mariposa*”.

Evidence for the effects of CLT is reflected in faster response times for cognates as compared to noncognates in picture naming [23, 26–31], as well as in word recognition and word translation [30, 32–34]. It has also been argued that cognates are processed as efficiently as monolinguals process mother tongue [35, 36]. Accordingly, cross-linguistic therapy effects with cognates in cases of bilingual aphasia have been examined. Roberts and Deslauriers [30] showed that highly proficient bilinguals with aphasia could better name cognates than non-cognates, and they also produced distinct error types for each target. Specifically, errors with cognates were no response and target description—the latter having a communicative value—whereas noncognates resulted in semantic errors as well as language switching errors [30].

Finally, although the evidence of a cognate effect in bilingual aphasia therapy is not unanimous [30, 37, 38], a generalization of therapy effects with cognates has been reported in a case of Spanish-English bilingual aphasia. Thus, Kohnert [37] reported cross-linguistic generalization of therapy effects from treated L1 (Spanish) to untreated L2 (English) for cognates only. Language treatment consisted of lexical semantic retrieval strategies such as word recognition, semantic association, and cueing [37]. Conversely, Kurland and Falcon [38] report an interference effect with cognates, following intensive language therapy with a semantic approach, in a case of a Spanish-English bilingual with chronic and severe expressive aphasia. This interference effect can be explained by reference to Abutalebi and Green's model [39]. The patient presented a lesion in the basal ganglia, a component of the corticosubcortical network sustaining the inhibition of the nontarget language; this network includes the left precentral cortex, the anterior cingulate, the inferior parietal lobule, and the basal ganglia [39].

Clangs, or homophones, also share phonological similarities with mother tongue words, but, unlike cognates, clangs refer to different concepts. The evidence of a clang effect in bilinguals is not convergent; thus, some authors argue that both orthographic and phonological similarity are required to facilitate word recognition [40, 41], whereas others claim that processing clangs imposed an extra cognitive load resulting from the inhibition of the nontarget semantic representation [42, 43]. In line with this claim, a recent functional connectivity study shows that healthy adults recruit a cognitive control network to process clangs [44]. The extent to which clangs may facilitate cross-linguistic therapy effects in bilinguals with aphasia has not yet been tested; however, the findings within healthy populations [42–45] suggest that clangs may become particularly difficult in cases of bilingual aphasia, given that brain damage entails decreased cognitive resources [46].

There is also a lack of convergence regarding CLTE with noncognates. Kurland and Falcon [38] reported successful CLTE for noncognates only, after therapy with a semantic approach. However, with a similar therapy approach, Kohnert [37] failed to report such an effect and instead found one with cognates. It is not easy to draw any conclusions given that such a small number of studies have compared cognates and noncognates, particularly because factors other than word type may have influenced therapy results in either language, including lesion location and extension as well as cross-linguistic similarities and differences.

#### 4.1.2. Structural Similarities and Differences across Languages

The degree of structural overlap across languages plays a major role in the potential for CLTE [19, 20]. For example, Goral et al. [47] described the case of a trilingual speaker with mild chronic aphasia, who was treated in English, (L2), first on morphosyntactic skills (i.e., pronoun and gender agreement) and then on language production rate. Measurements in the treated language (English) as well as in the two nontreated languages (Hebrew (L1), and French (L3)) were collected after each treatment block.

An improvement in pronoun and gender agreement in the treated language (L2) as well as in the nontreated L3 was observed following the treatment block on morphosyntactic skills in English. Also, there was an improvement in speech rate in English and in French following the second block, but no changes were observed in Hebrew. The authors concluded that selective CLT from L2 to L3 resulted from the structural similarities between English and French, as compared to a lack of similarity between English and Hebrew.

Similarly, Miertsch [48] administered semantic therapy in French (L3) to a trilingual participant with Wernicke's aphasia. Transfer was observed from L3 (French) to L2 (English), but not to L1 (German). These findings were interpreted as the result of structural similarities between French and English, as compared to French and German. However, there is also the possibility that the results in German reflect a plateau effect resulting from the fact that poststroke proficiency in German was higher than in the other two languages [48]. As discussed by Faroqui et al. [19], the years to come will yield more studies on the impact of cross-linguistic structural similarities and differences on CLTE.

#### 4.1.3. Pre-Morbid and Post-Morbid Proficiency in Either Language

A number of studies provide evidence for cross-linguistic transfer of therapy effects (CLTE) from the treated, less proficient second language, to the untreated and better preserved mother tongue. Kiran and Iakupova [49] administered semantic therapy in L2 (English) and measured naming on trained and untrained words both in L2 and L1 (Russian). Following therapy, the participant showed 100% accuracy in both treated and untreated items, thus reflecting successful CLTE. The authors [49] suggest that CLTE reflects the strengthened connections between the weaker (English) language and the stronger (Russian) language.

Likewise, CLTE was reported following intensive semantic therapy in L2 (English) in the case of a native Spanish bilingual individual with chronic, severe expressive aphasia [38], particularly on naming tasks. The authors argued that although CLTE from premorbid less proficient language (L2) to premorbid more proficient language (L1) had been successful, all gains considered that the patient benefited more from therapy in L1 than from therapy in L2.

There is evidence that balanced bilingualism contributes to CLTE [27, 50, 51], and, in cases of unbalanced bilingualism, transfer is observed from the less proficient language to the dominant language. Specifically, parallel recovery in both languages was observed in a premorbid balanced bilingual woman (Flemish, L1/Italian, L2) suffering from chronic aphasia after 2 weeks of picture-naming training through repetition and reading of names of pictures in L2 [51]. Similarly, Edmonds and Kiran [50] investigated the CLT of gains achieved following therapy with Semantic Feature Analysis to treat naming deficits by examining three English-Spanish bilinguals with aphasia, all of whom received a semantic therapy in Spanish (Participant 1) and in English and Spanish (Participants 2 and 3). Therapy effects were tested on treated items, untreated items, and translations; results showed that both within- and cross-language therapy effects were related to premorbid language proficiency. Specifically, Participant 1, a premorbid balanced bilingual, showed CLTE to the untreated English items, whereas Participants 2 and 3 (who were more proficient in English) showed within-language generalization to semantically related items, but no CLT to the untreated Spanish items. Moreover, though following treatment in Spanish, Participants 2 and 3 did not show any within-language generalization; they did show CLT to English, their dominant language. Thus, this data supports the idea that better CLTE is observed from the less proficient (L2) to the more proficient language (L1).

In another study, the authors [27] provided semantic therapy in Spanish to two Spanish-English bilinguals, one of them English dominant and the other one a balanced bilingual. Therapy in Spanish resulted in CLTE for both participants, whereas therapy in English was followed by CLTE in the balanced Spanish-English participant only.

Thus, some studies [27, 38, 49–51] provide evidence that premorbid proficiency in either language modulates CLTE, arguing that CLTE occurs more easily from a less proficient language to the dominant language in unbalanced bilinguals, whereas balanced bilingualism facilitates CLTE no matter which language is treated. Thus, it has been shown that the less proficient L2 relies upon the stronger L1 lexicon, whereas, at high proficiency levels, L1 and L2 lexicons are mostly overlapping [19, 52]. Nevertheless, it is difficult to draw a final conclusion, as some of these studies did not report poststroke proficiency states [27, 50].

A different point of view on the impact of proficiency is presented by Goral [53], who claims that it is postmorbid proficiency that determines the extent of CLTE. Evidence from four different case studies demonstrating successful CLTE with different patterns in multilingual participants with aphasia, included (a) CLTE in L1 (Hebrew) following treatment of L2 (English), (b) CLTE in L4 (German) following treatment of L5 (English), (c) CLTE in L3 (French) following treatment of the strongest language L2 (English), and (d) CLTE in L2 (German) following treatment of most recovered L3 (English). In all cases, CLTE occurred when the therapy was offered in the language with higher postmorbid proficiency, regardless of premorbid proficiency. This is also the case in the limited (only for cognates) CLTE in an L1 and L2 premorbidly highly proficient Spanish (L1) and English (L2) bilingual suffering from nonfluent aphasia reported by Kohnert [37]. This patient showed improvement after receiving therapy in both languages; however, CLTE was seen only when therapy was administered in the language with higher postmorbid proficiency (L1).

Similarly, Croft et al. [54] examined five English-Bengali bilinguals with aphasia and anomia, who received a phonological approach and a semantic cueing approach, both in L1 and L2. While phonological cueing resulted in no significant CLTE, semantic cueing led to CLTE for three out of five patients. In all cases, CLTE occurred only when therapy was offered in L1 [54]. In observing the data on the participants' aphasia profiles, one notes that, for all cases in which successful CLTE was reported, the language of therapy happened to be the stronger post-morbid language. As this postmorbid more proficient language also happened to be L1, the authors took these results as evidence for successful CLTE from L1 to L2, despite the fact that not all participants who were treated in L1 showed successful CLTE. Another case of unsuccessful CLT despite the balanced proficiency both at premorbid and postmorbid proficiency was reported by Abutalebi and colleagues [55]. Thus, no CLTE was observed following L2 treatment in a case of fluent aphasia. The patient was a highly proficient, balanced Spanish (L1) Italian (L2) bilingual, who had become severely anomic in both languages following aphasia, and involuntary language interference, was observed. Treatment in L2 was successful but did not show any CLTE. Unsuccessful CLTE in this case may result from the therapy approach chosen (phonological approach); however, another possibility is that involuntary language switching and unsuccessful CLTE resulted from damage to areas involved in cognitive control.

#### 4.1.4. Cognitive Control and Transfer of Therapy Effects

It has been shown that damage to the cognitive control circuit can prevent CLTE. However, there is also evidence that choosing an appropriate therapy approach (i.e., Switch Back Through Translation) can result in CLTE even when damage to the cognitive control circuit is observed [56]. This can be accomplished by implementing a strategy of translation of involuntary switches which allows bypassing the effects of impaired inhibitory abilities resulting from damage to the cognitive control circuit.

In the case reported by Abutalebi et al. [55], the Spanish (L1) and Italian (L2) bilingual anomic patient had damage to the left lenticular nucleus and surrounding areas. He showed selective L1 recovery at T0, and, when asked to name pictures in L2, he would unintentionally name in L1. However, after receiving therapy in L2, the selective pattern changed in favor of L2 and, thus, when asked to name in L1, he would unintentionally name in L2.

The change of selective recovery pattern and the fact that EM was unable to translate, together with the presence of a lesion within the cognitive control circuit, lead Abutalebi and colleagues [55] to conclude that EM's behavior supports the Dynamic Model on Recovery Patterns in Bilingual Aphasia, proposed by Green and Abutalebi [57]. According to this model [57], the same neural network supports L1 and L2 processing; however, the processing of the weaker language (usually L2) may as well involve the left prefrontal cortex, the basal ganglia, and the anterior cingulated cortex,as a function of proficiency level.

Based on Green and Abutalebi [57], one can argue that the recovery pattern will depend on the integrity of the circuits normally involved in language control; also, it may be hypothesized that damage to that circuit can affect CLTE. Thus, cognitive control encompasses controlling language selection, and its impairment may result in involuntary language mixing and language switching [56]. However, as previously discussed, the evidence shows that it is possible to compensate for this deficit by choosing an appropriate therapy approach, that can be designed by reference to a comprehensive model of bilingual language processing [56]. Precisely, CLTE can be triggered by stimulating both languages simultaneously in the context of a therapy approach that includes translation tasks, even when therapy is provided primarily in one language. Ansaldo et al. [56] reported the case of a Spanish-English bilingual who suffered from pathological language mixing, which caused alternation between Spanish (L1) and English (L2) utterances, in the context of communicating with monolingual Spanish speakers. The authors [56] analysed this behaviour within the framework of Green's model [46] and developed a procedure called SBTT (Switch Back Through Translation), based on the fact that translation from English to Spanish would provide an economic strategy to switch back to the target language, as opposed to inhibiting the nontarget (English) language, a lost ability resulting from brain damage to the language control circuit [56]. The therapy was primarily administered in Spanish and resulted in significant improvement in naming nouns and verbs in Spanish, but, moreover, CLTE to English was as well observed, both with nouns and verbs. Using translation may favour CLTE by stimulating cognitive processes that are common to the two languages of the bilingual individual (i.e., cognitive control of language selection). Further studies are required to explore this hypothesis in depth.

### 4.2. Promoting CLTE in Bilingual Aphasia Therapy: Main Clinical Implications of Research Findings

Despite the fact that more work is needed, research on CLTE in bilingual aphasia provides some cues as to the best approach of this clinical population.

In particular, the evidence suggests that language therapy focused on cognates facilitates CLTE. Thus, forming a list of cognates, consulting dictionaries developed for specific language pairs (e.g., Spanish-English: DOC—Dictionary of Cognates and the RDOC—Reverse Dictionary of Cognates [58, 59]) can help clinicians focus language therapy on stimuli with CLTE potential, communicative, and social relevance for the patients, their families, and caregivers. Furthermore, the MDOC project, which aims at joining the cognate matches for five language pairs (http://www.cognates.org/), will become an important resource in the management of bilinguals with aphasia. As for clangs, the evidence in healthy populations shows that their processing implies complex interactions with distinct semantic representations that share L1-L2 phonological forms, which may become particularly challenging for individuals with brain damage. Further research is required to shed light on this issue.

Regarding pre- or postmorbid proficiency, it is not easy to draw an absolute conclusion. Some studies [27, 38, 49–51] suggest that premorbid proficiency matters and that training the premorbid weaker language appears to facilitate CLTE, given that treating the weaker language has a greater effect on the stronger than the reverse. This has proven to be true for premorbidly unbalanced bilinguals and also for balanced bilinguals, who, after a stroke, showed an unbalanced language profile with distinct degrees of impairment in L1 and L2. On the other hand, other cases suggest that postmorbid proficiency is the determinant factor for successful CLTE [53]. Therefore, both premorbid and postmorbid proficiency should be considered when deciding the language of therapy, and, to do so, a thorough assessment of bilingual aphasia is a must.

Moreover, using translation as a CLT strategy may enhance the effects of therapy provided in one language to the untreated language. Translation equivalents are strongly linked, a factor that may facilitate CLTE. This approach may be particularly useful when damage excludes the cognitive control circuit, which supports the ability to switch between L1 and L2.

With respect to the anomia therapy approach, evidence suggests that Semantic Feature Analysis or a combination of this approach with phonological cueing may contribute to CLTE. Semantic Feature Analysis capitalizes on shared semantic representations across languages, and it has been shown to facilitate CLTE in bilinguals with aphasia [27]. Furthermore, the evidence with monolinguals shows that this approach triggers neuroplasticity in cases of severe anomia resulting from extensive brain damage [60].

Also, the impact of semantic and phonological approaches depending on the degree of L1-L2 cognate and clang density or global structural overlap needs to be explored. Hence, the evidence on healthy populations shows that processing structurally distant (i.e., unsimilar) languages entails greater cognitive demands [45]. Considering this evidence, it is likely that brain damage will hinder CLTE in bilinguals speaking distant languages, who suffer from aphasia.

Table 1 summarizes all studies discussed in Section 4.

## 5. Conclusion

Globalization imposes a number of challenges to the field of neurorehabilitation, including challenges in the clinical management of bilinguals with aphasia. In recent decades, the assessment and intervention techniques available to bilingual clinical populations have become a major clinical and research topic.

The study of intervention with bilingual aphasia populations has evolved from a descriptive perspective, mainly focused on case reports, to a neuropsychological and neurofunctional perspective, aimed at unveiling the cognitive and neural mechanisms underlying the behavioral patterns that characterize bilingual aphasia and its recovery. More and more, this avenue is focusing on disentangling the mechanisms that allow for transferring therapy effects from the treated to the untreated language. Most research has focused on anomia, the most widespread aphasia sign.

The literature suggests that cross-linguistic therapy effects are possible but depend on a number of factors. For example, both pre- and postmorbid proficiency factors can affect CLTE. Thus, while treating the premorbid weaker language can show CLTE benefits [27, 38, 49–51], cross-linguistic transfer of therapy effects are as well reported for eight cases whenever therapy is provided in the postmorbid stronger language or when proficiency after stroke is equivalent in both languages. Regarding therapy approach, the evidence from 16 studies reporting the type of therapy administered suggests that semantic approaches result in better CLTE than phonological approaches [54, 55]. Finally as for word types, cognates have better CLT potential than non-cognates [30, 37], but the cognate advantage disappears when cognitive control circuits are damaged [38]. This is the case probably because of reduced excitatory and inhibitory resources secondary to the damage in the cognitive control circuit. This impairment prevents correct selection among highly overlapping and competing lexical units (i.e., cognates). Green's Activation, Control and Resource Model [46, 61] assumes that lexical selection of the target word requires sufficient inhibitory (to suppress the non-target node) and excitatory resources (to activate the target node). Furthermore, 11 studies having reported CLT effects show no evidence suggesting that language distance could play a role on the potential for CLT in bilingual aphasia therapy. Thus, among indo-European languages, therapy effects can transfer across languages regardless of what language family they belong to the Indo-European family of languages [37, 47–49, 51, 53, 54].

Major developments in the field can be expected in the years to come. By combining clinical aphasiology, cognitive models of bilingualism, functional neuroimaging, and functional connectivity analysis it will be possible to better understand the mechanism that subserve CLT of therapy effects, and thus design bilingual aphasia therapy approaches accordingly. This will increase the probability of recovery from bilingual aphasia, while optimizing health care efficiency, in terms of resource allocation and training.

## Figures and Tables

**Table 1:** 

	Cognates	L1	L2	L3	Language family			Language proficiency			Therapy approach	Language of therapy	Successful transfer of therapy to untreated language
					L1	L2	L3	L1	L2	L3			
Roberts and Deslauriers (1999) [30]	×	French	English	NA	Roman	Germanic	NA	Pre-H	Pre-H	NA	NA	NA	NA

Kohnert (2004) [37]	×	Spanish	English	NA	Roman	Germanic	NA	Pre-H Post-I	Pre-H Post-L	NA	Lexical semantic retrieval strategies	L1	Cognates only

Kurland and Falcon (2011) [38]	×	Spanish	English	NA	Roman	Germanic	NA	Pre-H Post-I	Pre-I Post-L	NA	Semantic	L2	Noncognates, only

Goral et al. (2010) [47]	NA	Hebrew	Englis0068	French	Canaanite	Germanic	Roman	Pre-H Post-H	Pre-H Post-I	Pre-H Post-L	Morpho-syntactic skills and language production rate	L2	L3 only

Miertsch (2009) [48]	NA	German	English	French	German	Germanic	Roman	Pre-H Post-H	Pre-H Post-I	Pre-H Post-I	Semantic	L3	Only L2

Kiran and Iakupova (2011) [49]	NA	Russian	English	NA	Slavic	Germanic	NA	Pre-H Post-H	Pre-I Post-I	NA	Semantic	L2	L1

Marangolo et al. (2009) [51]	NA	Flemish	Italian	NA	Germanic	Roman	NA	Pre-H Post-I	Pre-H Post-I	NA	Picture-naming training	L2	yes

Edmonds and Kiran (2006) [50]/P1	NA	English	Spanish	NA	Germanic	Roman	NA	Pre-H Post-NR	Pre-I Post-NR	NA	Semantic feature analysis	L2	No

Edmonds and Kiran (2006) [50]/P2 and P3	NA	English	Spanish	NA	Germanic	Roman	NA	Pre-H Post-NR	Pre-I Post-NR	NA	Semantic feature analysis	L1 and L2	yes

Edmonds and Kiran (2006) [50], b/p1	NA	English	Spanish	NA	Germanic	Roman	NA	Pre-H Post-NR	Pre-I Post-NR	NA	Semantic feature analysis	L2 and L1	From L1 to L2 only

Edmonds and Kiran (2006) [50], b/p2	NA	Spanish	English	NA	Roman	Germanic	NA	Pre-H Post-NR	Pre-H Post-NR	NA	Semantic feature analysis	L1 and L2	From L1 to L2 only

Goral (2012) [53], P.1	NA	Hebrew	English	French	Canaanite	Germanic	Roman	Pre-H Post-H	Pre-H Post-I	Pre-H Post-L	Modified constraint-induced therapy	L2	L1

Goral (2012) [53], P.2	NA	Persian	German	English	Iranian	Germanic	Germanic	Pre-H Post-L	Pre-H Post-I	Pre-H Post-H	Modified constraint-induced therapy	L3, L1, and L2	From L2 to L3 only

Goral (2012) [53], P.3	NA	English	Hebrew	NA	Germanic	Canaanite	NA	Pre-H Post-H	Pre-I Post-I	NA	Modified constraint-induced therapy	L2	L1 but Negative

Goral (2012) [53], P.4	NA	Catalan	Spanish	French	Roman	Roman	Roman	Pre-H Post-H	Pre-H Post-H	Pre-I Post-I	Modified semantic feature analysis, sentence generate-on	L4: German, Pre-I, Post-I	L5: English Pre-I Post-I

Croft et al. (2011) [54]/P.1–5	NA	Bengali	English	NA		Germanic	NA	Post-H for 3 Ps	Post-L for 2 Ps	NA	Phonological and semantic cueing	L1 and L2	For semantic cueing only

Abutalebi et al. (2009) [55]	NA	Spanish	Italian	NA	Roman	Roman	NA	Pre-H Post-L	Pre-H Post-L	NA	Phonological training	L2	No
